# Epithelial‐Mesenchymal Plasticity in the D‐Meso‐Sonobe Mesothelioma Cell Line: A Putative Model of Epithelial–Mesenchymal Transition in Mesothelioma

**DOI:** 10.1111/1759-7714.70091

**Published:** 2025-05-21

**Authors:** Hiroshi Okubo, Yuki Hanamatsu, Chiemi Saigo, Sonobe Hiroshi, Tamotsu Takeuchi

**Affiliations:** ^1^ Department of Pathology and Translational Research Gifu University Graduate School of Medicine Gifu Japan; ^2^ Center for One Medicine Innovative Translational Research; COMIT Gifu University Gifu Japan; ^3^ The United Graduate School of Drug Discovery and Medical Information Sciences Gifu University Japan; ^4^ Department of Diagnostic Pathology National Hospital Organization, Fukuyama Medical Center Fukuyama Japan

**Keywords:** D‐Meso‐Sonobe, epithelial‐to‐mesenchymal transition, mesothelioma, YAP, Zeb1

## Abstract

Epithelial‐mesenchymal transition (EMT) plays a crucial role in carcinogenesis, including mesothelioma. D‐Meso‐Sonobe is a deciduoid‐type mesothelioma cell line with morphological features similar to those of epithelioid cells. Here, we report that D‐Meso‐Sonobe cells exhibit spindle cell mesenchymal features under continuous confluent culture conditions. The spindle cell mesenchymal D‐Meso‐Sonobe expresses zinc finger E‐box‐binding homeobox 1 (Zeb1), which is a master regulator of EMT in the nucleus. Xenoplanted D‐Meso‐Sonobe cells expressed nuclear Zeb1 and yes‐associated protein at the cancer invasion front and focally expressed integrin subunit alpha V and actin alpha 2, which are molecular phenotypes acquired by EMT in mesothelioma. Subsequent RNA sequencing revealed that lysyl oxidase like 1 (LOXL1) was more highly expressed in cultured spindle mesenchymal D‐Meso‐Sonobe cells than in epithelioid cells. LOXL1 immunoreactivity was observed at the invasive front of xenoplanted D‐Meso‐Sonobe cells. The epithelial‐mesenchymal plasticity of D‐Meso‐Sonobe cells may be applicable for the development of candidate molecular agents targeting EMT in mesothelioma.

## Graphical abstract

EMT plasticity of D‐Meso‐Sonobe cells D‐Meso‐Sonobe cells exhibited epithelioid features in passage culture at approximately 60% confluence (upper left), but became spindle‐cell mesenchymal cells after continuous full confluent culturing (upper right). LOXL1 immunoreactivity was found at the invasion front near muscle cells, while minimum staining was observed in the non‐invasion front (lower).

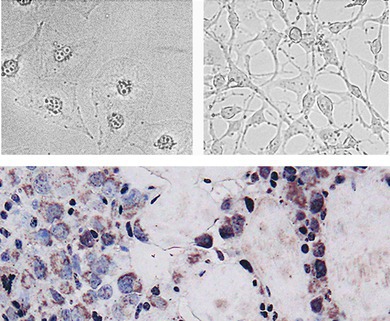

## 1 Introduction

Mesothelioma is a highly aggressive malignant neoplasm with limited therapeutic options and is often resistant to chemotherapy [1]. Even with the latest combination immunotherapies, the median overall survival of patients with mesothelioma is approximately 18 months [2, 3], thus warranting further therapeutic strategies.

The epithelial–mesenchymal transition (EMT) promotes migration, invasion, metastasis, stemness, resistance to chemotherapy, and immune evasion during cancer progression [4]. In the genesis of mesothelioma, EMT plays a central role in the epithelioid–sarcomatoid continuum [1]. Therefore, interruption of EMT may be a therapeutic option for patients with mesothelioma [5]. From another perspective, EMT in mesotheliomas is a suitable model for studying cancerous EMT processes [6]. Here, we propose a simple cell culture‐based model that reflects the EMT process in mesotheliomas.

## 2 Materials and Methods

### 2.1 Cells and Culture

A detailed characterization of the deciduoid‐type mesothelioma cell line D‐Meso‐Sonobe has been previously provided [7]. D‐Meso‐Sonobe cells were cultured in Dulbecco's modified Eagle's medium‐high glucose (4500 mg/L) (Sigma‐Aldrich, St. Louis, MO) with 10% fetal bovine serum. D‐Meso‐Sonobe cells were passaged at approximately 60% confluence. D‐Meso‐Sonobe cells were cultured to confluence and for 36 h to obtain mesenchymal spindle cell features. Cell images were acquired using CytoWatcher (ATTO, Tokyo, Japan).

### 2.2 Cell Lysates and Immunoblotting

Cytoplasmic and nuclear fractions of D‐Meso‐Sonobe cell lysates were obtained using a LysoPure extraction kit (Fujifilm Wako Pure Chemical, Osaka, Japan).

Immunoblotting was performed according to the method described by Towbin et al. with modifications as previously described [8, 9, 10]. Briefly, the cell lysates were electrophoresed on sodium dodecyl sulfate‐polyacrylamide gels and electroblotted onto polyvinylidene difluoride membranes (Immobilon‐P transfer membranes; Millipore, Bedford, MA). Membranes were blocked with Block Ace (blocking milk; Yukijirushi, Sapporo, Japan) and incubated with antibodies. Detailed characterization of rabbit anti‐zinc finger E‐box‐binding homeobox 1 (Zeb1) has been previously described [11, 12]. For immunodetection, horseradish peroxidase‐conjugated anti‐rabbit secondary antibody (cat. no. 7074; Cell Signaling Technology, Danvers, MA) and an ultrasensitive horseradish peroxidase substrate (TaKaRa Bio, Kusatsu, Japan). Images were obtained using an Invitrogen iBright 1500 gel imaging system (Thermo Fisher Scientific, Waltham, MA). After stripping the immunoreactivity, the membranes were incubated with murine anti‐glyceraldehyde‐3‐phosphate dehydrogenase (GAPDH) antibody (cat no. 60004‐1‐Ig; Proteintech, Chicago, IL) or rabbit laminin a/c antibody (cat no. 10298‐1‐AP; Proteintech), followed by incubation with horseradish peroxidase‐conjugated anti‐mouse secondary antibody (cat. no. 7076; Cell Signaling Technology Inc.) or anti‐rabbit antibody (cat. no. 7074; Cell Signaling Technology Inc.) to evaluate the input proteins.

### 2.3 Immunofluorescent Cytochemistry Staining

Immunofluorescence staining was performed as previously described procedure [13]. Briefly, the cells were fixed in 4% paraformaldehyde, permeabilized in 0.2% Triton X‐100/100 mM glycine/PBS (pH 7.4), and blocked in 3% BSA/PBS at room temperature. Rabbit anti‐Zeb1 and secondary Alexa Fluor 488 goat anti‐rabbit IgG (H+L) (cat. A32731; Life Technologies, Carlsbad, CA) were diluted in a blocking solution and incubated for 1 h or 45 min at room temperature. Nuclei were stained with 4′,6‐diamidino‐2‐phenylindole (DAPI) (Nacalai Tesque, Kyoto, Japan). Images were acquired using a confocal laser scanning microscope (TCS SP8, Leica, Germany) and processed using Leica Application Suite X software, as previously described [14].

### 2.4 Immunohistochemical Staining

The detailed procedure for D‐Meso‐Sonobe xenoplantation followed by immunohistochemical staining has been previously reported [7, 15]. Severe combined immunodeficiency‐Nonobese diabetic (SCID‐NOD; NOD.CB17‐*Prkdc* ^*scid*^/J) mice were purchased from Charles River Laboratories. D‐Meso‐Sonobe cells (5 × 10^6^) were subcutaneously injected into the thigh soft tissue of 10‐week‐old male mice. The tissue specimens were fixed in 10% buffered formalin and embedded in paraffin. The rabbit antibody to yes‐associated protein (YAP) was obtained from Cell Signaling Technology (cat. no. 4912). The rabbit antibodies against Integrin Subunit Alpha V (ITGAV) (cat. no. 27096‐1‐AP), and actin alpha 2 (ACTA2) (cat. no. 14395‐1‐AP) were purchased from Proteintech. The rabbit antibody against LOXL1 (cat. no. GTX114532) was purchased from GeneTex (Irvine, CA). Tissues were immunostained with antibodies using the ImmPRESS polymerized reporter enzyme staining system (Vector Laboratories, Burlingame, CA).

All animal experiments were conducted at Gifu University following the guidelines for animal experimentation and adhering to the Japanese Law for the Humane Treatment and Management of Animals. The experimental protocol was approved by the Animal Care Committee of the Gifu Graduate School (approval no. AG‐P‐N‐20220087 and ‐20220147).

### 2.5 RNA‐Sequencing (RNA‐Seq)

Cultured D‐Meso‐Sonobe cell specimens—comprising two epithelioid and two spindle‐cell mesenchymal types—were subjected to RNA‐seq. Total RNA was extracted using the NucleoSpin RNA kit (MACHEREY‐NAGEL, Düren, Germany). RNA integrity was confirmed with a BioAnalyzer (Agilent Technologies) RNA 6000 Nano Kit, and all samples had an RNA integrity number (RIN) greater than 9.6. RNA‐Seq was outsourced to Novogene Co. Ltd., which followed standard protocols. Briefly, mRNA was purified from total RNA using poly‐T oligo‐attached magnetic beads to construct the sequencing library. The resulting raw reads were aligned to the human genome sequence (GRCh38), and significance was defined as a false discovery rate (FDR) < 0.05.

## 3 Results

### 3.1 Continuous Confluent Cell Culture Altered the Morphology of D‐Meso‐Sonobe Cells From Epithelioid to Spindle‐Cell

**FIGURE 1:**
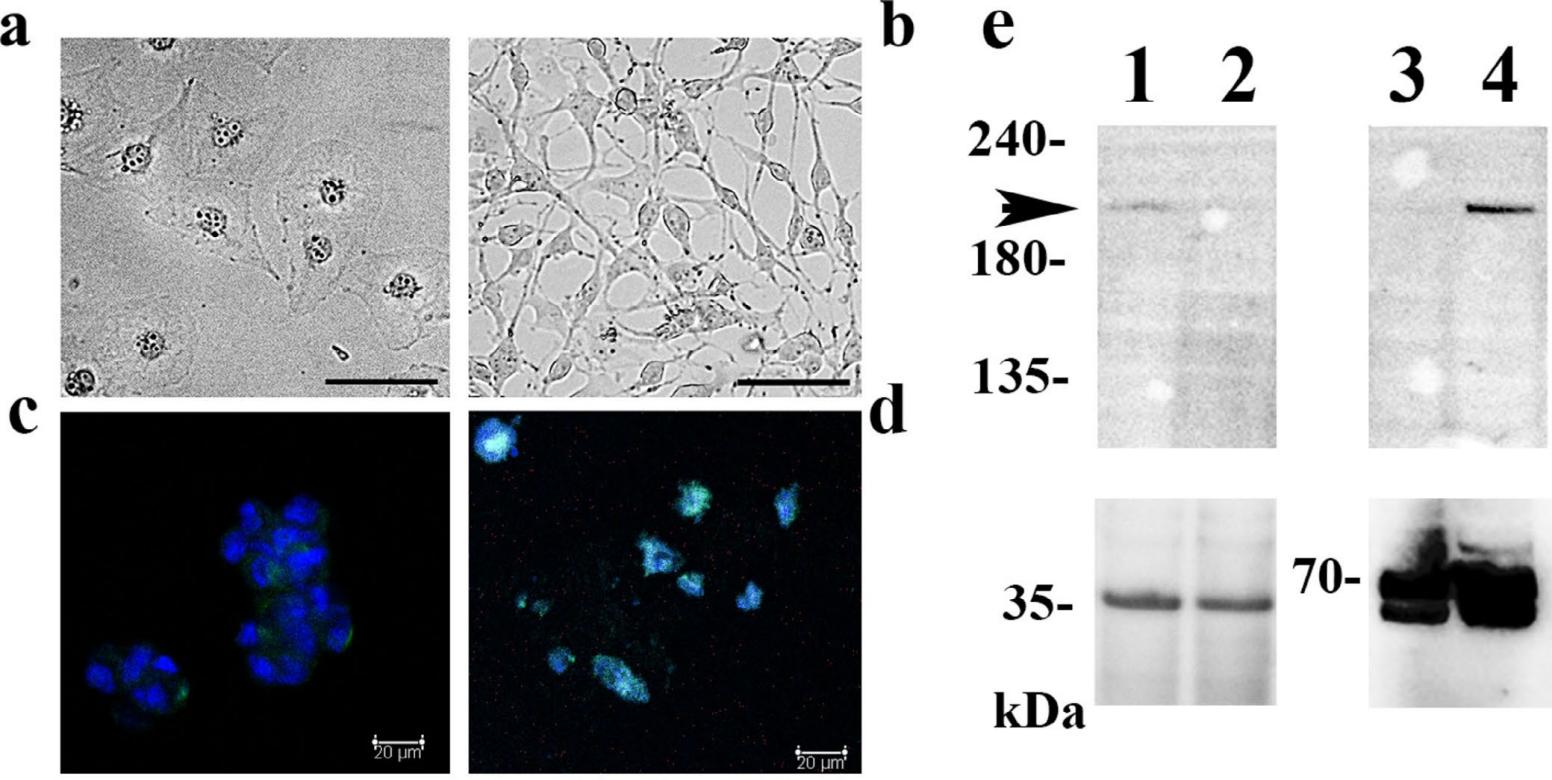
EMT plasticity of D‐Meso‐Sonobe cells in vitro. D‐Meso‐Sonobe cells exhibited epithelioid features in passage culture at approximately 60% confluence (a), but became spindle‐cell mesenchymal cells after continuous full confluent culturing (b). Weak cytoplasmic green Zeb1 immunoreactivity was observed in the epithelioid D‐Meso‐Sonobe cells (c). In contrast, nuclear Zeb1 immunoreactivity, cyan signal merging of nuclear blue DAPI staining, and green Zeb1 immunoreactivity were observed in mesenchymal D‐Meso‐Sonobe cells (d). Scale bar, 50 μm (a and b) and 20 μm (c and d). An approximately 200‐kDa Zeb1 protein band indicated by an arrow was found in the cytoplasmic lytic fraction of the epithelioid D‐Meso‐Sonobe cells (lane 1) but not in the spindle‐cell mesenchymal D‐Meso‐Sonobe cells (lane 2). In contrast, the Zeb1 protein band was found in the nuclear lytic fraction of the spindle cell mesenchymal D‐Meso‐Sonobe cells (lane 4) but not in the epithelioid D‐Meso‐Sonobe cells (lane 3). The GAPDH (lanes 1 and 2) and laminin a/c (lanes 3 and 4) loading bands are shown in the lower columns. The raw immunoblot data are shown in Figure S1.

The D‐Meso‐Sonobe cells were passaged at approximately 60% confluence. Under these cell culture conditions, D‐Meso‐Sonobe cells exhibited epithelioid (Figure S1) features (Figure 1a), i.e., cuboidal, oval, or polygonal shape, abundant cytoplasm, and ovoid to round nuclei. Incidentally, we found that D‐Meso‐Sonobe cells exhibited spindle‐cell mesenchymal features under continuous full‐confluent cell culture for 36 h (Figure 1b). The spindle‐cell mesenchymal D‐Meso‐Sonobe cells became epithelioid after passage culture.

### 3.2 Spindle‐Cell Mesenchymal D‐Meso‐Sonobe Cells Expressed Zeb1 Protein in the Nucleus

Immunofluorescence staining showed that epithelioid D‐Meso‐Sonobe cells barely expressed Zeb1 immunoreactivity in the cytoplasm (Figure 1c), whereas spindle‐cell mesenchymal D‐Meso‐Sonobe cells strongly expressed nuclear Zeb1 immunoreactivity (Figure 1d). A weak Zeb1 protein band was detected in the cytoplasmic fraction of epithelioid, but not spindle‐cell, D‐Meso‐Sonobe cells. The Zeb1 protein band was observed in the nuclear fraction of cell lysates from spindle‐cell mesenchymal D‐Meso‐Sonobe cells (Figure 1e).

### 3.3 Differentially Expressed Molecules Between Epithelioid and Spindle‐Shaped Mesenchymal D‐Meso‐Sonobe Cells

**FIGURE 2:**
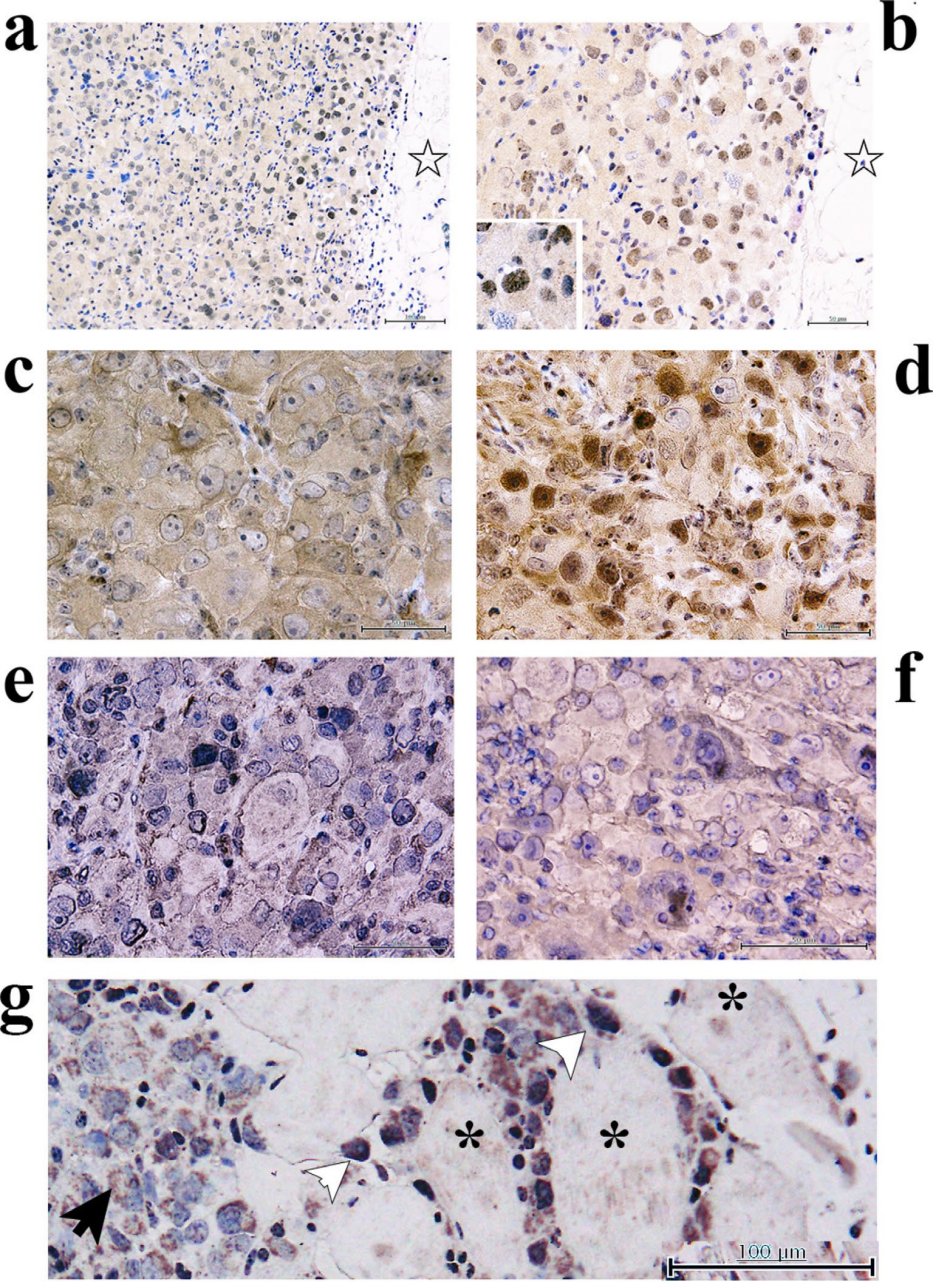
Immunohistochemical staining of xenoplanted D‐Meso‐Sonobe tumor cells. Zeb1 immunoreactivity was barely found in xenoplanted tumor cells (a and b). Furthermore, nuclear Zeb1 immunoreactivity was found in various tumor cells at the cancer invasion front (indicated by ☆ in a and b). Cytoplasmic yes‐associated protein (YAP) immunoreactivity was found in center of the xenoplanted tumor (c), whereas nuclear YAP immunoreactivity was also found in tumor invasion front (d). Integrin Subunit Alpha V and Actin alpha 2 immunoreactivities were focally found in the xenoplanted tumor (e and f, respectively). LOXL1 immunoreactivity was found at the invasion front near muscle cells (indicated by white arrowhead), while minimum staining was observed in the non‐invasion front (indicated by black arrow). Scale bars represent 100 μm (a and g) and 50 μm (b–f).

We first evaluated the expression of EMT molecules, which have been associated with prognosis in patients with mesothelioma, including Zeb1, YAP, ITGAV, and ACTA2. Zeb1 immunoreactivity was weakly detected in the cytoplasm of xenoplanted D‐Meso‐Sonobe cells at the center of the tumors (Figure 2a,b). By contrast, strong Zeb1 immunoreactivity was observed in the nuclei of D‐Meso‐Sonobe cells at the invasion front of the xenoplanted tumors (Figure 2b). Cytoplasmic YAP immunoreactivity predominated at the center of the xenoplanted tumor (Figure 2c), whereas nuclear YAP immunoreactivity was observed at the invasion front (Figure 2d). Immunoreactivities for ITGAV and ACTA2 were focal in the xenoplanted tumors (Figure 2e,f).

Subsequently, we investigated whether transcriptional events drive the EMT plasticity of D‐Meso‐Sonobe cells using RNA‐seq. The data quality and representative results from the RNA‐seq analysis are summarized in Tables S1 and S2, respectively. Notably, the transcript for the lysyl oxidase‐like 1 (LOXL1) gene was considerably more abundant in cultured spindle‐shaped mesenchymal D‐Meso‐Sonobe cells than in epithelioid cells. LOXL1 immunoreactivity was also observed at the invasion front of xenoplanted D‐Meso‐Sonobe tumors (Figure 2g), coinciding with the accumulation of Zeb1 immunoreactivity in the nucleus. LOXL1 is believed to play a role in various carcinogenesis processes by promoting EMT. Taken together, we suggest that the EMT plasticity of D‐Meso‐Sonobe cells is at least partially driven by transcriptional events.

## 4 Discussion

EMT is a crucial pathobiological process in which cancer cells lose their epithelioid features and gain a mesenchymal phenotype, leading to metastasis and drug resistance [4, 16]. The generation of simple models reflecting cancerous EMT is important for the development of molecular agents against EMT.

In this study, we demonstrated that epithelioid D‐Meso‐Sonobe cells turned into spindle‐cell mesenchymal cells after continuous, fully confluent culturing. Notably, spindle‐cell mesenchymal D‐Meso‐Sonobe cells recovered their epithelioid features after simple cell passage. These findings indicate that D‐Meso‐Sonobe cells have a plasticity resembling that of EMT in mesothelioma.

The results of the immunohistochemical staining of xenoplanted D‐Meso‐Sonobe tumors also support the idea that D‐Meso‐Sonobe cells constitute a suitable EMT model. Nuclear Zeb1 immunoreactivity was observed at the xenoplanted tumor invasion front. Recently, Guo et al. [17] reported that Zeb1 shuttles between the cytoplasm and nucleus in lung cancer. They revealed that lung cancer cells exhibited cytoplasmic Zeb1, which maintained an epithelial expression pattern by interrupting actin cytoskeletal assembly. At invasion sites, Zeb1 is transported to the nucleus, relieving cytoskeletal inhibition and initiating EMT gene expression [17]. Cytoplasmic Zeb1 also inhibits the nuclear translocation of YAP (herein referring to YAP1), a key mediator of EMT in the Hippo pathway, to extend the initiation of EMT [17, 18]. ITGAV and ACTA2 are representative EMT‐related proteins in mesothelioma [6]. LOXL1 is believed to promote EMT in various types of carcinogenesis [19, 20, 21]. In the present RNA‐seq analysis, transcripts of *Zeb1, YAP, integrin subunit alpha V, actin alpha 2*, and *LOXL1* were approximately 1.23‐, 1.05‐, 1.46‐, 3.03‐, and 5.27‐fold higher, respectively, in spindle‐shaped mesenchymal cells than in epithelioid D‐Meso‐Sonobe cells. Alternations in the subcellular localization of Zeb1 and/or YAP may contribute to the transcriptional upregulation of EMT‐related molecules. Taken together, the xenoplanted D‐Meso‐Sonobe cells also reflected EMT plasticity in mesothelioma.

In conclusion, D‐Meso‐Sonobe cells may be useful for screening for candidate molecular agents against cancerous EMT.

## Author Contributions

All authors had full access to the data in the study and take responsibility for the integrity of the data and the accuracy of the data analysis. Conceptualization: S.H. and T.T. Methodology: H.O., Y.H., and C.S. Investigation: H.O. Formal analysis: Y.H. and T.T. Resources: S.H. and T.T. Writing – original draft: C.S. and T.T. Writing – review and editing: S.H. and T.T. Visualization: H.O. and T.T. Supervision: S.H. and T.T. Funding acquisition: Y.H. and T.T.

## Ethics Statement

All animal experiments were conducted at Gifu University following the guidelines for animal experimentation and adhering to the Japanese Law for the Humane Treatment and Management of Animals. The experimental protocol was approved by the Animal Care Committee of the Gifu Graduate School (approval no. AG‐P‐N‐20220087 and ‐20220147).

## Conflicts of Interest

The authors declare no conflicts of interest.

## Supporting information


**Figure S1.** Immunoblotting raw data.

**Table S1.** Independently cultured epithelioid D‐Meso‐Sonobe cells, designated as E1 and E2, and spindle‐shaped mesenchymal D‐Meso‐Sonobe cells, designated as S1 and S2, were subjected to RNA‐seq. Note the low error and high effective rates, 0.01 (< 0.1) and nearly or more than 98%, respectively. Raw reads: total amount of reads in raw data. Raw data: (Raw reads)*(sequence length). For paired‐end sequencing like PE150, the sequencing length equals 150. Effective: (Clean reads/Raw reads)*100%. Error: base error rate. Q20, Q30: (Base count with Phred value > 20 or 30)/(total base count). GC: (G & C base count)/(total base count).

**Table S2.** Representative differentially expressed transcripts between the epithelioid (E1 and E2) and spindle‐shape mesenchymal (S1 and S2) D‐Meso‐Sonobe cell groups are presented. Approximately 100 transcripts, selected based on a false discovery rate (FDR) threshold of < 0.05, along with their transcripts per million (TPM) values, are listed.

## Data Availability

The data that support the findings of this study are available from the corresponding author upon reasonable request.
