# Supplementary figures and images for: Epithelial‐Mesenchymal Plasticity in the D‐Meso‐Sonobe Mesothelioma Cell Line: A Putative Model of Epithelial–Mesenchymal Transition in Mesothelioma
Source: Thorac Cancer. 2025 May 21;16(10):e70091. doi: 10.1111/1759-7714.70091 (PMC12093248; doi:10.1111/1759-7714.70091)

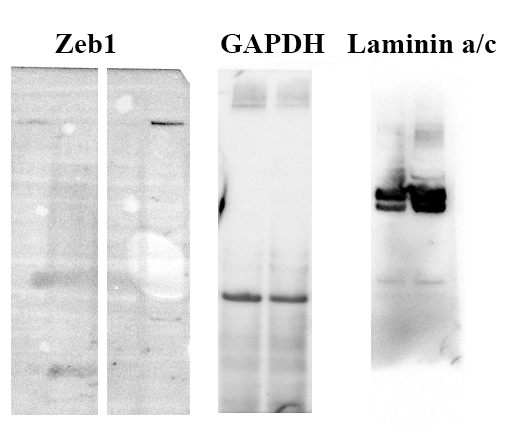

Supplement: Supplementary file 1 — Figure S1. Immunoblotting raw data. [file TCA-16-e70091-s001.tif]

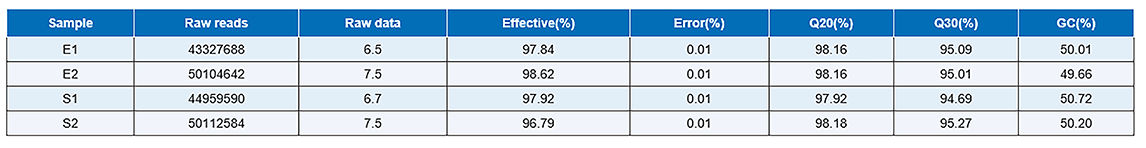

Supplement: Supplementary file 2 — Table S1. Independently cultured epithelioid D‐Meso‐Sonobe cells, designated as E1 and E2, and spindle‐shaped mesenchymal D‐Meso‐Sonobe cells, designated as S1 and S2, were subjected to RNA‐seq. Note the low error and high effective rates, 0.01 (< 0.1) and nearly or more than 98%, respectively. Raw reads: total amount of reads in raw data. Raw data: (Raw reads)*(sequence length). For paired‐end sequencing like PE150, the sequencing length equals 150. Effective: (Clean reads/Raw reads)*100%. Error: base error rate. Q20, Q30: (Base count with Phred value > 20 or 30)/(total base count). GC: (G & C base count)/(total base count). [file TCA-16-e70091-s003.png]
